# Consensus statements on the definition, diagnosis, and management of oligoprogressive disease: results of an expert survey of Kazakhstan Cancer Society

**DOI:** 10.37349/etat.2026.1002392

**Published:** 2026-08-05

**Authors:** Dilyara Kaidarova, Indira Omarova, Samat Kaldarbekov, Nazgul Omarbayeva, Maia Dzhugashvili, Elena Ulrikh, Dina Alisheva, Bakhadyr Bereketov, Ilya Tsimafeyeu, Timur Mitin

**Affiliations:** Chinese Academy of Medical Sciences & Peking Union Medical College, China; Fondazione Policlinico Universitario Agostino Gemelli IRCCS, Italy; ^1^Asfendiyarov Kazakh National Medical University, Almaty 050012, Republic of Kazakhstan; ^2^Kazakhstan Cancer Society, Almaty 050040, Republic of Kazakhstan; ^3^Karaganda Medical University, Karaganda 100012, Republic of Kazakhstan; ^4^Outpatient Department, Zhambyl Regional Multidisciplinary Oncology and Surgery Center, Taraz 080000, Republic of Kazakhstan; ^5^Breast Cancer Department, Kazakh Institute of Oncology and Radiology, Almaty 050022, Republic of Kazakhstan; ^6^Department of Radiation Oncology, GenesisCare, Madrid 28010, Spain; ^7^N.N. Petrov National Medical Research Center of Oncology, Saint Petersburg 197758, Russian Federation; ^8^Almaty Oncology Center, Almaty 050054, Republic of Kazakhstan; ^9^Department of Urological Oncology, Kazakh Institute of Oncology and Radiology, Almaty 050022, Republic of Kazakhstan; ^10^Bureau for Cancer Research, New York, NY 10032, USA; ^11^Department of Radiation Oncology, Knight Cancer Institute, Oregon Health and Science University, Portland, OR 97239, USA

**Keywords:** oligoprogressive disease, management, consensus, Kazakhstan Cancer Society

## Abstract

**Aim:**

Oligoprogressive disease (OPD) has emerged as a clinically relevant and common scenario in oncology, reflecting progression in a limited number of metastatic sites while systemic therapy continues to control the majority of disease. Despite its increasing recognition, the definition, diagnostic approach, and management of OPD remain poorly standardized.

**Methods:**

A multidisciplinary expert panel from Kazakhstan and international faculty, including ten medical oncologists, radiation, and surgical oncologists, participated in a structured survey. The questionnaire consisted of 15 items divided into three domains: definition of OPD, diagnostic evaluation, and treatment strategies. Responses were analyzed, and consensus was defined as ≥ 70% agreement, relative agreement as 50–69%, and lack of consensus as < 50%.

**Results:**

Consensus was reached that OPD is defined as progression in a limited number of lesions while other sites remain controlled, regardless of lesion size or localization. No consensus was achieved regarding the maximum number of progressive lesions, with experts divided between “≤ 5 lesions” and “any number amenable to local therapy.” Imaging with CT or PET-CT was considered sufficient. Biopsy and molecular testing were recommended only in selected contexts. Systemic therapy continuation during OPD was endorsed if effective, with strong support for local therapy, particularly stereotactic ablative radiotherapy (SBRT/SABR). Divergence remained regarding the management of repeat OPD.

**Conclusions:**

This Kazakhstan Cancer Society consensus defines the fundamental clinical features of OPD and provides practical recommendations for diagnosis and treatment. Comparison with international guidelines reveals broad alignment on systemic continuation and local therapy, as well as persistent variation regarding lesion number, biopsy, and repeat OPD management.

## Introduction

The concept of oligoprogressive disease (OPD) has gained increasing attention in recent years, driven by the widespread use of targeted therapies and immune checkpoint inhibitors [1, 2]. While these treatments often achieve durable systemic disease control, selective resistance may develop in a limited number of lesions [3]. The biological mechanisms underlying such resistance are heterogeneous and may include the emergence of new mutations in distinct tumor clones, activation of alternative signaling pathways, receptor overexpression, or the development of an immunosuppressive tumor microenvironment [4–6]. Consequently, the concept of applying targeted local therapies to sites of oligoprogression has emerged as a rational therapeutic strategy aimed at eradicating resistant clones while maintaining the benefit of ongoing systemic treatment.

The clinical management of OPD presents several challenges. Key decisions include whether to continue or modify systemic therapy, whether to obtain tissue for molecular characterization of resistance mechanisms, and which local treatment modalities such as surgery, radiotherapy, or ablative techniques should be employed.

Major international societies, including the European Society for Medical Oncology (ESMO), the European Society for Radiotherapy and Oncology (ESTRO), and the American Society for Radiation Oncology (ASTRO), recognize OPD as a distinct clinical entity. However, significant variability persists in its definition and management, largely due to the limited availability of prospective clinical evidence. To date, only a small number of randomized trials have specifically evaluated the role of local therapies in oligoprogressive settings [7–10]. As a result, current recommendations are predominantly based on expert consensus, which is inherently influenced by institutional experience, availability of technologies, and regional practice patterns.

In this context, the development of national and regional consensus guidelines is critically important, particularly to ensure that recommendations are both evidence-informed and adaptable to local healthcare systems and resource availability.

In Kazakhstan, no formal consensus on the management of OPD had previously been established. To address this gap, we convened a multidisciplinary panel of experts in medical, surgical, and radiation oncology. This manuscript presents the results of a structured consensus process, identifies areas of agreement and controversy, and contextualizes the Kazakhstan Cancer Society recommendations within the broader landscape of international guidelines.

## Materials and methods

This consensus was developed specifically for Kazakhstan, reflecting the national clinical context, availability of diagnostic and therapeutic resources, and existing practice patterns. While the recommendations are intended to harmonize national practice, they are also presented here to contribute to the broader international discussion of OPD management.

The study employed a Delphi methodology to develop national consensus recommendations on the definition, diagnosis, and management of OPD. The methodology consisted of three sequential phases: (1) review of the available scientific evidence and international consensus statements; (2) a structured face-to-face meeting of the expert panel to discuss the evidence, identify key areas of uncertainty, and finalize the survey questionnaire; and (3) an anonymous Delphi survey to establish consensus using predefined agreement criteria.

The expert panel consisted of 10 senior specialists from tertiary cancer centers in Kazakhstan, the United States, Spain, and Russia. The panel included three medical oncologists, three radiation oncologists, and four surgical oncologists, all with more than 10 years of clinical experience and active participation in multidisciplinary tumor boards. Importantly, these experts constitute the Kazakhstan Cancer Society working group responsible for developing national oncology guidelines in this field and therefore represent the complete national expert panel for OPD. In Delphi studies, panel quality and expertise are considered more important than panel size, and homogeneous expert panels of approximately 8–23 participants are widely regarded as methodologically appropriate.

The questionnaire was developed by a steering committee after reviewing contemporary literature, international consensus statements, and clinical practice guidelines The final survey included 15 structured questions, grouped into three thematic domains: (1) definition of OPD (six questions), (2) diagnostic evaluation (three questions), and (3) treatment strategies (six questions). Each item was posed as a multiple-choice question, with options reflecting both prevailing international definitions and locally relevant clinical practices.

A questionnaire was distributed to all panel members. During the Delphi process, experts were instructed to answer independently, without discussion among the panel, to avoid group influence on responses. Responses were submitted anonymously via a secure online form, which ensured confidentiality while allowing quantitative analysis. Panelists were encouraged to provide free-text comments where they wished to elaborate on their answers.

Finally, responses were compiled and analyzed by the coordinating committee.

Survey responses were analyzed using descriptive statistics using the IBM SPSS Statistics Base v22.0 software (SPSS, Inc., Chicago, Illinois, USA). For each question, the number of responses for each option was recorded and presented as both absolute counts (*n*/*N*) and percentages. Agreement percentages were calculated by dividing the number of experts selecting a given response by the total number of respondents. Consensus was prospectively defined as agreement by ≥ 70% of panelists, relative agreement as 50–69%, and lack of consensus as < 50%. Because the objective of the study was to summarize expert opinions rather than test statistical hypotheses, no inferential statistical analyses were performed.

This study was based exclusively on an anonymous survey of expert opinions and did not involve human participants, patient data, biological specimens, or animal research. Therefore, institutional review board (IRB) or ethics committee approval were not required under applicable national regulations. Participation by all experts was voluntary and all of them provided written informed consent.

## Results

### Statement 1. Definition of oligoprogressive disease

All experts agreed that OPD should be defined as progression in a limited number of lesions with otherwise controlled systemic disease, confirming this as the fundamental conceptual framework (Table 1). Regarding the maximum number of progressive lesions, responses were split: half of the panel supported the threshold of up to five lesions, while the other half preferred a more flexible definition based on the technical feasibility of local therapy. Eighty percent of respondents endorsed the view that OPD may occur both in the setting of ongoing effective systemic therapy and at the time of primary progression, while 2 (20%) restricted oligoprogression only to situations of systemic control. Most experts (8; 80%) stated that the anatomical localization of metastases should not influence the definition, and all agreed that lesion size was not a determinant. Finally, while six experts did not consider RECIST 1.1 measurability mandatory for OPD definition, four favored applying these criteria, leading to relative agreement.

**Table 1:** Definition of oligoprogressive disease.

**Question**	**Main responses**	**Consensus status**
Is oligoprogressive disease correctly defined as progression in a limited number of lesions, with control over the others?	Yes: 10 (100%)	Consensus
What number of progressive lesions is acceptable for oligoprogressive disease?	≤ 5 lesions: 5 (50%) Any number amenable to local therapy: 5 (50%)	No consensus
Should oligoprogressive disease only occur during effective systemic therapy, or also at primary progression?	Both scenarios: 8 (80%) Only during effective therapy: 2 (20%)	Consensus
Does localization of metastases affect oligoprogressive disease definition?	Not relevant: 8 (80%) Relevant: 2 (20%)	Consensus
Does lesion size matter for oligoprogressive disease definition?	Not relevant: 10 (100%)	Consensus
Should oligoprogressive lesions be measurable according to RECIST 1.1 criteria?	Not required: 6 (60%) Required: 4 (40%)	Relative agreement

RECIST: Response Evaluation Criteria in Solid Tumors.

### Statement 2. Diagnosis

On diagnostic evaluation, the panel showed relative agreement regarding the need for biopsy, with most experts recommending morphological verification of oligoprogressive lesions in most cases, while four preferred a more selective approach (Table 2). Similarly, molecular analysis was endorsed by 6 (60%) only in select cases, with the remainder favoring routine use. In contrast, strong consensus was achieved for imaging: 9 (90%) of experts agreed that both CT (computed tomography scan) and PET-CT (positron emission tomography-CT) are appropriate modalities for assessing OPD, with only 1 (10%) of panelist preferring PET-CT alone.

**Table 2:** Diagnosis.

**Question**	**Main responses**	**Consensus status**
Should oligoprogressive lesions be morphologically verified (biopsy)?	Yes: 6 (60%) No/exceptional: 4 (40%)	Relative agreement
Should histology be obtained for molecular analysis?	Select cases: 6 (60%) Always: 4 (40%)	Relative agreement
Which imaging methods are standard for the diagnosis of oligoprogressive disease?	CT or PET-CT: 9 (90%) PET-CT only: 1 (10%)	Consensus

CT: computed tomography scan; PET-CT: positron emission tomography-CT.

### Statement 3. Treatment strategies

The panel reached strong consensus on the principle that systemic therapy should be continued during OPD if it remains effective, with 8 (80%) agreement (Table 3). With respect to local therapy, relative consensus was observed: 6 (60%) of experts supported a context-dependent approach, while 4 (40%) recommended prioritizing local treatment in all cases. Regarding the choice of local therapy, 6 (60%) stated that the modality should depend on lesion localization, whereas 4 (40%) explicitly preferred radiation therapy. Nevertheless, when radiation therapy was chosen, 8 (80%) agreed that stereotactic ablative radiation therapy (SBRT/SABR) should be the regimen of choice. Finally, in the management of repeat OPD, opinions diverged, with 6 (60%) of experts supporting a switch in systemic therapy after local treatment, while 4 (40%) of endorsed continuing the same systemic therapy.

**Table 3:** Treatment strategies.

**Question**	**Main responses**	**Consensus status**
Should local therapies be prioritized in oligoprogressive disease occurring under effective systemic therapy?	Context-dependent: 6 (60%) Always: 4 (40%)	Relative agreement
Which local therapy is preferred for oligoprogressive disease?	Any modality depending on site: 6 (60%) Radiotherapy (SBRT/SABR): 4 (40%)	Relative agreement
If radiation therapy is chosen, is there a preferred regimen?	SBRT/SABR: 8 (80%) No preference: 2 (20%)	Consensus
Should systemic therapy be continued during oligoprogressive disease if effective?	Yes: 8 (80%) No: 2 (20%)	Consensus
For repeat oligoprogression, what management strategy is appropriate?	Local + systemic switch: 6 (60%) Local + continue systemic therapy: 4 (40%)	Relative agreement

SBRT: stereotactic body radiation therapy; SABR: stereotactic ablative body radiotherapy.

## Discussion

This consensus exercise provides a structured framework for the definition and management of OPD in Kazakhstan. Several findings warrant emphasis. The unanimous agreement that OPD should be defined as progression in a limited number of lesions with otherwise controlled disease provides a solid foundation for clinical practice. Importantly, lesion size and anatomical localization were not considered relevant determinants. The absence of consensus regarding the maximum number of progressive lesions reflects two distinct conceptual approaches to defining OPD. Experts supporting a threshold of up to five lesions based their opinion primarily on the eligibility criteria used in prospective randomized trials of local ablative therapy, in which patients with no more than five metastatic lesions were typically enrolled. From this perspective, maintaining a numerical threshold preserves consistency with the existing evidence base and facilitates comparison across clinical studies. In contrast, experts favoring a definition based on technical feasibility argued that the biological behavior of OPD cannot be adequately captured by an arbitrary lesion count. Advances in imaging modalities, such as PET/CT scan and whole-body MRI, have increased the detection of small metastatic lesions that would have remained occult using conventional imaging. Consequently, the number of detectable lesions may depend more on imaging sensitivity than on the underlying biology of the disease. Furthermore, continuous improvements in stereotactic radiotherapy, image-guided ablative procedures, and surgical techniques have expanded the number of lesions that can be safely treated in selected patients. These experts therefore considered the feasibility of delivering radical local therapy to all progressive sites to be more clinically meaningful than adherence to a fixed numerical threshold. This divergence reflects the current international debate and underscores the need for prospective studies evaluating whether biological characteristics or technical treatability should define OPD.

Diagnostic evaluation achieved consensus on imaging, with CT and PET-CT both considered appropriate. The role of biopsy and molecular testing remained unsettled, consistent with international guidelines that increasingly recommend molecular profiling but acknowledge the practical challenges of implementation. In terms of management, systemic therapy continuation during oligoprogression was strongly endorsed, aligning with real-world practice and retrospective evidence. Local therapies, particularly SBRT/SABR, were prioritized, although not universally required. The absence of consensus on the management of repeat limited progression underscores the need for prospective clinical trials.

Consensus statements are not developed in isolation but are inevitably shaped by local realities. In Kazakhstan, contrast-enhanced CT remains the standard imaging technique because it is widely accessible and provides sufficient information for most treatment decisions. PET-CT is increasingly available at tertiary referral centers and is considered particularly valuable when conventional imaging yields equivocal findings, when occult sites of disease are suspected before local ablative therapy, or when confirmation of true oligoprogression may alter management. Nevertheless, its routine use for every patient with suspected OPD is limited by availability and healthcare costs. Similarly, the panel favored a selective rather than universal approach to biopsy and molecular testing. Histological confirmation is considered appropriate when imaging findings are atypical, when a second primary malignancy or a benign process cannot be excluded, or when transformation of tumor histology is suspected. Molecular testing may be clinically informative in selected patients, particularly when identification of resistance mechanisms could influence subsequent systemic therapy or enrollment in clinical trials. However, because actionable resistance alterations remain incompletely characterized for many solid tumors and comprehensive molecular profiling is not uniformly available throughout Kazakhstan, routine biopsy and molecular testing for every patient with OPD were not considered justified. This pragmatic approach reflects current national practice while remaining consistent with international recommendations that advocate individualized use of advanced diagnostic techniques.

When compared with international societies, the Kazakhstan consensus broadly aligns with ESTRO, ASTRO, ABC, and EAU in emphasizing systemic continuation and local therapy as central strategies (Table 4). However, differences exist in definitions of lesion number and in the role of biopsy [11–14]. Canadian and Chinese expert groups similarly endorse systemic continuation but tend to favor stricter lesion thresholds and more routine molecular profiling [15, 16]. Importantly, most published consensus statements have addressed oligometastatic disease or combined oligometastatic and OPD under a unified framework. The present work is distinct in that it focuses exclusively on OPD, recognizing it as a separate clinical scenario that arises under the selective pressure of systemic therapy. This provides more tailored guidance for clinicians and highlights the need for further research directed specifically at OPD.

**Table 4:** Comparison of consensus of Kazakhstan Cancer Society with international guidelines.

**Comparison**	**Kazakhstan consensus**	**ESTRO/ASTRO and others [11–14]**	**Canadian/Chinese guidelines [15, 16]**
Scope	Focus exclusively on oligoprogressive disease	Primarily oligometastatic disease; some mention oligoprogression	Primarily oligometastatic disease, occasionally include oligoprogressive disease
Definition	Limited progression under systemic control; lesion size/localization not relevant	Limited number of metastases; oligoprogressive disease sometimes acknowledged but not central	Oligometastatic disease defined as ≤ 3–5 lesions; oligoprogressive disease treated as subset
Number of lesions	No consensus (≤ 5 vs. any treatable number)	Typically ≤ 3–5 lesions	≤ 3–5 lesions
Diagnosis	CT or PET-CT sufficient; biopsy and molecular testing in selected cases	Imaging standard; biopsy increasingly encouraged	Imaging standard; molecular testing emphasized
Treatment	Continue systemic therapy if effective; local therapy prioritized, SBRT/SABR favored	Local therapy + systemic continuation recommended	Local therapy + systemic continuation standard; stricter lesion thresholds
Repeat oligo-progression	No consensus (systemic switch vs. continued systemic with local therapy)	Rarely specified	Early systemic switch sometimes favored

CT: computed tomography scan; PET-CT: positron emission tomography-CT; SBRT: stereotactic body radiation therapy; SABR: stereotactic ablative body radiotherapy.

Importantly, present consensus should not be interpreted as an alternative to international guidelines but rather as their adaptation to a national healthcare environment. Consensus recommendations inevitably reflect not only the available evidence but also local expertise, technology, and healthcare infrastructure. By explicitly incorporating these practical considerations, the Kazakhstan Cancer Society consensus complements existing international guidance and illustrates how global recommendations can be translated into routine clinical practice. The remaining areas of disagreement, particularly the definition of oligoprogression and the management of repeat OPD, highlight the urgent need for prospective randomized studies capable of generating higher-level evidence for future guideline development.

This consensus has several limitations. First, as an expert consensus, the recommendations are based on specialist opinion rather than prospective clinical evidence. Second, although the panel included all national experts responsible for developing oncology guidelines in Kazakhstan and international collaborators, the recommendations may still reflect local clinical practice patterns and healthcare infrastructure, which could limit their generalizability to other healthcare systems. Third, the proposed recommendations have not yet been prospectively validated, and their impact on clinical outcomes remains to be demonstrated in future studies. Finally, the consensus process did not include patient representatives or patient-reported perspectives, which are increasingly recognized as important components of shared decision-making, particularly when balancing the benefits and risks of local treatment strategies. These limitations underscore that the present recommendations should complement, rather than replace, individualized multidisciplinary decision-making and provide a framework for future prospective research.

In conclusion, the Kazakhstan Cancer Society consensus provides practical guidance for the definition, diagnosis, and management of OPD. The recommendations support the use of standardized terminology, emphasize appropriate patient selection for local therapy, and promote multidisciplinary decision-making while acknowledging persistent areas of uncertainty, including lesion number, biopsy, and the management of repeat oligoprogression.

Beyond summarizing current expert opinion, this consensus provides a framework for harmonizing routine oncology practice across Kazakhstan and may serve as the foundation for future national clinical guidelines on OPD. As new evidence from prospective clinical trials becomes available, these recommendations should be updated to further optimize patient care and align national practice with evolving international standards.

## Abbreviations

ASTRO: American Society for Radiation Oncology
CT: computed tomography scan
ESTRO: European Society for Radiotherapy and Oncology
OPD: oligoprogressive disease
PET-CT: positron emission tomography-computed tomography scan

## Declarations

### Availability of data and materials

The data supporting the findings of this study are available from the corresponding author upon reasonable request.

### Copyright

© The Author(s) 2026.
